# Vitamin D deficiency and long-term risk of epilepsy or seizure in type 2 diabetes: a multicenter real-world cohort study

**DOI:** 10.3389/fnut.2026.1910295

**Published:** 2026-08-07

**Authors:** Ming Yew, Ping-Hsin Liu, Ping-Hsun Feng

**Affiliations:** 1 Department of Anesthesiology, Chi Mei Medical Center, Tainan, Taiwan; 2 Department of Anesthesiology, E-Da Hospital, I-Shou University, Kaohsiung, Taiwan; 3 Department of Anesthesiology, Chi Mei Hospital, Liouying, Tainan, Taiwan

**Keywords:** epilepsy, propensity score matching, real-world data, seizure, type 2 diabetes mellitus, vitamin D deficiency

## Abstract

**Background:**

Type 2 diabetes mellitus (T2DM) is associated with an increased risk of seizures and epilepsy; however, clinically measurable factors contributing to this risk remain unclear. Given the high prevalence of vitamin D deficiency (VDD) in T2DM and its potential effects on neuronal excitability, this study aimed to investigate the association between VDD and the long-term risk of incident epilepsy or seizure in adults with T2DM.

**Methods:**

We conducted a retrospective propensity score–matched cohort study using the TriNetX Global Collaborative Network. Adults with T2DM and a recorded 25-hydroxyvitamin D measurement between 2013 and 2023 were classified as having VDD (<20 ng/mL) or normal vitamin D levels (≥30 ng/mL). The primary outcome was incident epilepsy or seizure during an analytic window extending up to 12 years after the index date. The secondary outcomes included epilepsy subtypes, antiepileptic medication use, and all-cause mortality.

**Results:**

After matching, 135,329 patients were included in each cohort. VDD was associated with a higher risk of incident epilepsy or seizure than normal vitamin D status (1.10% vs. 0.78%; HR, 1.37; 95% CI, 1.26–1.48; *p* < 0.001). Similar associations were observed for focal (HR, 1.40; *p* < 0.001), generalized (HR, 1.42; *p* = 0.003), and intractable epilepsy (HR, 1.92; *p* < 0.001). VDD was also associated with greater antiepileptic medication use (HR, 1.25; *p* < 0.001) and higher all-cause mortality (HR, 1.47; *p* < 0.001). The association with the primary outcome remained stable across the sensitivity analyses and was generally consistent in the subgroup analyses stratified by sex and age. Exploratory exposure-gradient analyses showed progressively higher risk estimates with worsening vitamin D status.

**Conclusion:**

Among adults with T2DM, VDD was associated with a higher long-term risk of incident epilepsy or seizure, particularly intractable epilepsy. These findings suggest that VDD may serve as a clinically relevant risk marker; however, they do not establish causality or demonstrate that vitamin D supplementation prevents epilepsy or seizure.

## 1 Introduction

Type 2 diabetes mellitus (T2DM) is one of the most prevalent chronic diseases worldwide and is accompanied by a wide range of neurological and metabolic complications (1–4). Patients with T2DM have an elevated burden of seizures and epilepsy, attributable in part to recurrent hypoglycemia, cerebral microvascular disease, cerebrovascular events, autonomic and metabolic dysregulation, and chronic neuroinflammation (5–10). These risk factors may coexist and accumulate over time, making seizure-related outcomes an important but relatively under-recognized neurological complication in this population (8, 11, 12). Therefore, identifying the factors associated with this elevated risk is of clinical interest.

Vitamin D deficiency (VDD) is highly prevalent among patients with T2DM and may reflect a combination of inadequate sunlight exposure, adiposity, chronic inflammation, renal dysfunction, and altered metabolic health (13–15). Beyond its classical role in bone and mineral metabolism, vitamin D exerts neuromodulatory effects by regulating intracellular calcium homeostasis, neurotrophin expression, and anti-inflammatory and antioxidant pathways, which are biologically relevant to neuronal excitability and seizure susceptibility (16, 17). These observations raise the possibility that low vitamin D status may be associated with a higher risk of epilepsy, particularly in T2DM, in which VDD is common and seizure risk is already elevated.

However, existing evidence is limited and largely indirect. Most clinical studies have examined the reverse relationship, reporting a high prevalence of VDD among patients already diagnosed with epilepsy, partly related to long-term use of antiseizure medications (18–20). Mendelian randomization studies evaluating genetically predicted 25(OH)D levels and epilepsy risk have reported inconsistent or largely null findings (21–23). However, such studies (21–23) did not directly determine whether clinically measured VDD precedes incident epilepsy or seizure in real-world patients. Few longitudinal cohort studies have examined this temporal relationship, and none have specifically focused on patients with T2DM, in whom both VDD and seizure-related risk factors are common.

To address this gap, we conducted a large, multinational, propensity score–matched cohort study using real-world data to examine the association between VDD and the long-term risk of incident epilepsy and seizure in adults with T2DM. We further assessed epilepsy subtypes, antiepileptic drug use, mortality, and possible exposure-gradient relationships to characterize the robustness and consistency of this association.

## 2 Materials and methods

### 2.1 Study design and data source

We conducted a retrospective, propensity score–matched cohort study using the TriNetX Global Collaborative Network, a federated platform aggregating de-identified electronic health records from more than 150 healthcare organizations across multiple regions. The TriNetX platform has been extensively used in peer-reviewed studies across a broad range of clinical disciplines (24–27). Because only aggregated, de-identified data were analyzed, the requirement for individual informed consent was waived. The study was approved by the Institutional Review Board of Chi Mei Medical Center and conducted in accordance with the Declaration of Helsinki.

### 2.2 Exposure classification

We identified adults aged ≥18 years with type 2 diabetes mellitus (T2DM) who had a recorded serum or plasma 25-hydroxyvitamin D [25(OH)D] measurement between January 1, 2013, and December 31, 2023. T2DM was defined using the ICD-10-CM code E11. Vitamin D status was based on the first qualifying 25(OH)D measurement during the study period. VDD was defined as a serum or plasma 25(OH)D level <20 ng/mL, whereas the control cohort comprised patients with levels ≥30 ng/mL. Laboratory data within the network are harmonized to common clinical concepts and measurement units. However, information regarding assay manufacturers, analytical platforms, calibration procedures, and local quality-control practices was unavailable; therefore, we could not confirm that 25(OH)D assays were analytically standardized across participating healthcare organizations. The index date was defined as the date of the first qualifying vitamin D measurement that met the exposure definition. Eligible patients were required to have at least one healthcare encounter within 5 years before the index date to ensure adequate capture of baseline data. A 5-year look-back was further applied to confirm persistent rather than transient vitamin D status: patients with VDD who had any prior measurement ≥20 ng/mL and controls who had any prior measurement <30 ng/mL were excluded from the study.

### 2.3 Exclusion criteria and landmark analysis

Patients were excluded if they had a diagnosis of epilepsy or recurrent seizures (ICD-10-CM G40) or any recorded antiepileptic medication use before or during the 1-year period after the index date to reduce the likelihood of including patients with pre-existing epileptic disorders or early events temporally unrelated to vitamin D status. Patients who died within 1 year after the index date were also excluded, consistent with the 1-year landmark design used to reduce early reverse causation and survivorship bias in the study.

Additional exclusions were applied to reduce confounding by conditions that may substantially affect vitamin D metabolism, nutritional status, seizure risk, or outcome ascertainment. Patients were excluded if they had end-stage renal disease, stage 4 or 5 chronic kidney disease, dependence on renal dialysis, human immunodeficiency virus infection, liver fibrosis or cirrhosis, celiac disease, Crohn’s disease, short bowel syndrome, or pancreatic diseases before the index date. Patients with acute perturbations within 1 month before the index date (pregnancy, recent critical care, acute kidney failure, and sepsis), which can transiently depress 25(OH)D levels, were also excluded. We additionally excluded patients with major neurological conditions that independently predispose them to seizures, including intracranial injury, CNS neoplasms, encephalitis or myelitis, anoxic brain injury, cerebral palsy, dementia, and Alzheimer’s disease. The diagnostic codes used to define the exclusion criteria are summarized in Supplementary Table 1.

### 2.4 Propensity score matching

Patients in the VDD and control cohorts were matched 1:1 using greedy nearest-neighbor matching with a caliper width equal to 0.1 times the pooled standard deviation of the logit of the propensity score. Propensity scores were estimated using a logistic model incorporating demographics, comorbidities, medications, healthcare utilization, and laboratory values. We specifically included factors known to independently influence seizure risk in diabetes, including diabetes-related complications and prior hypoglycemia, because both can provoke seizures irrespective of vitamin D levels and would confound the association. Missing laboratory values were not imputed. Covariate balance was evaluated using standardized mean differences, with a value <0.10 indicating an acceptable balance. The codes used for all the matching variables are detailed in Supplementary Table 2.

### 2.5 Study outcomes

The primary outcome was incident epilepsy or seizure, defined using ICD-10-CM code G40, during an analytic window extending up to 12 years after the index date. Secondary outcomes assessed over the same period included epilepsy subtypes (focal, generalized, and intractable epilepsy), antiepileptic medication use, and mortality. Follow-up began after the completion of the 1-year landmark period and continued until the first occurrence of the outcome of interest, death, loss to follow-up, or the end of the study window, whichever occurred first.

Two control outcomes were prespecified: secondary hyperparathyroidism, a biologically plausible positive control related to VDD, and acute appendicitis, a negative control with no expected association with the vitamin D status. These outcomes were used to contextualize the specificity of the observed associations and assess potential residual confounding or surveillance-related artifacts. Subsequent low 25(OH)D levels during follow-up were evaluated to assess exposure durability, and the occurrence of at least one follow-up healthcare encounter was compared between groups to examine potential surveillance bias. The diagnostic codes for all outcomes are provided in Supplementary Table 3.

### 2.6 Sensitivity, subgroup, and multivariable analyses

Robustness was assessed using four sensitivity models, each addressing a distinct potential source of bias. Model I was restricted to patients who survived the follow-up period to evaluate whether the association persisted independent of differential mortality between groups. Model II was restricted to patients with at least one recorded follow-up encounter, ensuring comparable opportunities for outcome ascertainment and reducing potential detection bias. Model III excluded patients with prior hypoglycemia because hypoglycemia is a recognized seizure precipitant that may confound the association independently of vitamin D status. Model IV excluded patients whose vitamin D status crossed the pre-specified exposure threshold during follow-up, thereby reducing time-varying exposure misclassification.

Prespecified subgroup analyses were conducted by age (18–65 and >65 years) and sex, with interaction terms used to assess potential effect modification. As a complementary analysis independent of propensity score matching, we fitted a multivariable Cox proportional hazards model in the full unmatched cohort, adjusting for sex, age, race, hypertension, dyslipidemia, overweight/obesity, chronic kidney disease, ischemic heart disease, nicotine dependence, cerebrovascular disease, alcohol-related disorders, hypoglycemia, glycemic control, and liver disease.

### 2.7 Exposure-gradient analysis

To explore the exposure-gradient relationship beyond the primary VDD analysis, we performed additional matched comparisons for severe VDD (<10 ng/mL) and vitamin D insufficiency (20–29.9 ng/mL), each against normal vitamin D status (≥30 ng/mL), using the same propensity score matching strategy and outcome definitions as in the primary analysis. Progressively higher risk estimates across vitamin D insufficiency, VDD, and severe VDD were considered consistent with an exposure-gradient pattern. A formal trend test was not performed because this function was not available within the TriNetX analytic platform.

### 2.8 Statistical methods

The primary analysis used a cause-specific Cox proportional hazards framework. Hazard ratios (HRs) with 95% confidence intervals (CIs) were estimated, and survival was compared using Kaplan–Meier curves with the log-rank test. The proportional hazards assumption was evaluated using Schoenfeld residuals. *E*-values were computed for the primary outcome to assess the sensitivity to potential unmeasured confounding. Analyses were performed using the TriNetX Analytics platform. Statistical significance for the primary endpoint was defined using a two-sided α level of 0.05. All secondary, control, sensitivity, subgroup, and exposure-gradient analyses were treated as exploratory, with no adjustment for multiple comparisons.

## 3 Results

### 3.1 Patient selection and baseline characteristics

Prior to matching, 166,173 patients with VDD and 259,188 controls with normal vitamin D levels were eligible. Before matching, the proportions of missing laboratory values in the VDD and control cohorts were 18.0% and 22.5% for glucose, 26.6% and 32.6% for hemoglobin, 29.4% and 32.1% for albumin, and 34.6% and 38.2% for HbA1c, respectively. The two cohorts were imbalanced across several baseline characteristics (Table 1). The VDD group was younger (mean age, 56.0 vs. 65.0 years; SMD, 0.584), had a lower proportion of White patients (51.8% vs. 70.3%), and had a higher proportion of Black patients (25.5% vs. 12.9%). The groups also differed in the prevalence of disorders of lipoprotein metabolism, overweight or obesity, hypertension, neoplasms, and nicotine dependence (all SMD > 0.10). After 1:1 propensity score matching, 135,329 patients remained in each group. The baseline characteristics were well balanced between the groups, with all standardized mean differences <0.10. The mean age was similar between the VDD and control groups (59.7 vs. 59.8 years), as were the proportions of women (59.4% vs. 59.8%) and White patients (59.2% vs. 59.1%).

**TABLE 1:** Baseline characteristics of patients with vitamin D deficiency and normal vitamin D status before and after propensity score matching.

Variables	Before matching			After matching		
	VDD group (*n* = 166,173)	Control group (*n* = 259,188)	SMD	VDD group (*n* = 135,329)	Control group (*n* = 135,329)	SMD
Patient characteristics						
Age at index (years)	56.0 ± 16.7	65.0 ± 13.9	0.584	59.7 ± 14.8	59.8 ± 14.8	0.004
Female	97617 (58.7)	161495 (62.3)	0.073	80416 (59.4)	80957 (59.8)	0.008
BMI > 30 kg/m^2^	42936 (25.8)	90652 (35.0)	0.200	37800 (27.9)	38197 (28.2)	0.007
White	86105 (51.8)	182239 (70.3)	0.386	80112 (59.2)	79952 (59.1)	0.002
Black or African American	42388 (25.5)	33363 (12.9)	0.325	26656 (19.7)	27043 (20.0)	0.007
Asian	6937 (4.2)	14313 (5.5)	0.063	6347 (4.7)	5984 (4.4)	0.013
Comorbidities and health services						
Factors influencing health status and contact with health services	124569 (75.0)	197772 (76.3)	0.031	101317 (74.9)	101616 (75.1)	0.005
Essential (primary) hypertension	95435 (57.4)	166032 (64.1)	0.136	81224 (60.0)	81677 (60.4)	0.007
Disorders of lipoprotein metabolism and other lipidemias	85461 (51.4)	165745 (63.9)	0.255	75942 (56.1)	76319 (56.4)	0.006
Overweight and obesity	61944 (37.3)	70839 (27.3)	0.214	45001 (33.3)	45188 (33.4)	0.003
Neoplasms	36325 (21.9)	71989 (27.8)	0.137	32323 (23.9)	32092 (23.7)	0.004
Ischemic heart diseases	22680 (13.6)	43041 (16.6)	0.083	20234 (15.0)	19989 (14.8)	0.005
Obstructive sleep apnea	20577 (12.4)	30206 (11.7)	0.022	16533 (12.2)	16307 (12.1)	0.005
Anemias	18708 (11.3)	30214 (11.7)	0.013	15143 (11.2)	15169 (11.2)	0.001
Chronic kidney disease	16425 (9.9)	33688 (13.0)	0.098	14611 (10.8)	14493 (10.7)	0.003
Nicotine dependence	19359 (11.7)	17966 (6.9)	0.163	13180 (9.7)	13400 (9.9)	0.005
Diseases of liver	13699 (8.2)	19662 (7.6)	0.024	11055 (8.2)	10844 (8.0)	0.006
COPD	10551 (6.3)	16189 (6.2)	0.004	8935 (6.6)	8946 (6.6)	0.000
Cerebrovascular diseases	10054 (6.1)	18872 (7.3)	0.049	8895 (6.6)	8831 (6.5)	0.002
T2DM with kidney complications	9570 (5.8)	18304 (7.1)	0.053	8373 (6.2)	8229 (6.1)	0.004
T2DM with neurological complications	8367 (5.0)	13763 (5.3)	0.012	7096 (5.2)	6916 (5.1)	0.006
COVID-19	6752 (4.1)	10602 (4.1)	0.001	5404 (4.0)	5405 (4.0)	0.000
T2DM with ophthalmic complications	6006 (3.6)	8670 (3.3)	0.015	4908 (3.6)	4887 (3.6)	0.001
Alcohol related disorders	4361 (2.6)	3845 (1.5)	0.081	2874 (2.1)	2820 (2.1)	0.003
T2DM with hypoglycemia	2460 (1.5)	3327 (1.3)	0.017	1928 (1.4)	1851 (1.4)	0.005
Malnutrition	1946 (1.2)	2695 (1.0)	0.013	1537 (1.1)	1493 (1.1)	0.003
Medications						
Metformin	52531 (31.6)	79368 (30.6)	0.021	42115 (31.1)	41903 (31.0)	0.003
Insulins and analogues	40334 (24.3)	49981 (19.3)	0.121	30445 (22.5)	30314 (22.4)	0.002
Vitamin D	32296 (19.4)	47926 (18.5)	0.024	25091 (18.5)	25086 (18.5)	0.000
GLP-1 analogues	10524 (6.3)	18447 (7.1)	0.031	8964 (6.6)	8829 (6.5)	0.004
SGLT2 inhibitors	7491 (4.5)	15062 (5.8)	0.059	6738 (5.0)	6670 (4.9)	0.002
Laboratory data						
Glucose < 70 mg/dL	10913 (6.6)	13102 (5.1)	0.065	8192 (6.1)	8185 (6.0)	0.000
Hemoglobin > 12 g/dL	110054 (66.2)	160298 (61.8)	0.091	88358 (65.3)	88757 (65.6)	0.006
Albumin > 3.5 g/dL	111653 (67.2)	171529 (66.2)	0.021	90519 (66.9)	90780 (67.1)	0.004

Data are presented as number (%) or mean ± standard deviation. Vitamin D deficiency was defined as serum 25-hydroxyvitamin D < 20 ng/mL, and normal vitamin D status was defined as serum 25-hydroxyvitamin D ≥ 30 ng/mL. Standardized mean differences <0.10 were considered acceptable for covariate balance. COPD, chronic obstructive pulmonary disease; GLP-1, glucagon-like peptide-1; SGLT2, sodium-glucose cotransporter 2; SMD, standardized mean difference; T2DM, type 2 diabetes mellitus; VDD, vitamin D deficiency.

### 3.2 Primary and secondary outcomes

The mean follow-up durations were 5.0 years in the VDD group and 4.9 years in the control group, and the corresponding median durations were 4.5 and 4.3 years. VDD was associated with a higher risk of incident epilepsy or seizure than normal vitamin D status (1,486 [1.10%] vs. 1,050 [0.78%]; HR, 1.37; 95% CI, 1.26–1.48; *p* < 0.001) (Table 2). The proportional hazards assumption was not violated for the primary outcome based on the Schoenfeld residual test (*p* = 0.951). Although the relative association was statistically significant, the absolute incidence difference was small (0.32 percentage points). The primary outcome *E*-value was 2.08 for the point estimate and 1.83 for the lower 95% confidence interval bound, meaning that an unmeasured confounder would need to be associated with both VDD and incident epilepsy or seizure by risk ratios of at least 2.08 each, beyond the measured covariates, to fully explain the observed association. Although the absolute incidence was low in both groups, similar associations were observed for focal epilepsy (HR, 1.40; *p* < 0.001), generalized epilepsy (HR, 1.42; *p* = 0.003), and intractable epilepsy (HR, 1.92; *p* < 0.001), with the largest relative estimate observed in intractable epilepsy. VDD was also associated with greater antiepileptic medication use (HR, 1.25; *p* < 0.001) and higher all-cause mortality (9.43% vs. 6.18%; HR, 1.47; *p* < 0.001).

**TABLE 2:** Association between vitamin D deficiency and risks of epilepsy-related outcomes and mortality in patients with type 2 diabetes.

Outcome	VDD group (*n* = 135,329)	Control group (*n* = 135,329)	HR (95% CI)	*P*-value	Absolute risk difference (percentage points)
	Events (%)	Events (%)			
Primary outcome					
Epilepsy or seizure	1,486 (1.10)	1,050 (0.78)	1.37 (1.26–1.48)	<0.001	0.32
Secondary outcomes					
Focal epilepsy	353 (0.26)	243 (0.18)	1.40 (1.19–1.65)	<0.001	0.08
Generalized epilepsy	175 (0.13)	118 (0.09)	1.42 (1.13–1.80)	0.003	0.04
Intractable epilepsy	87 (0.06)	43 (0.03)	1.92 (1.33–2.76)	<0.001	0.03
Antiepileptics	25,138 (18.58)	20,294 (15.00)	1.25 (1.23–1.27)	<0.001	3.58
Mortality	12,766 (9.43)	8,366 (6.18)	1.47 (1.43–1.51)	<0.001	3.25

Data are presented as number of events (%). Absolute risk difference was calculated as the event percentage in the VDD group minus that in the control group and is expressed in percentage points. CI, confidence interval; HR, hazard ratio; VDD, vitamin D deficiency.

### 3.3 Control and supportive outcomes

The prespecified control outcomes showed an expected pattern. The positive control, secondary hyperparathyroidism, was more frequent in the VDD group (HR, 1.50; *p* < 0.001), whereas the negative control, acute appendicitis, was not significantly associated with VDD (HR, 1.08; *p* = 0.298) (Table 3). Follow-up healthcare encounters were slightly less frequent in the VDD group (HR, 0.92; *p* < 0.001), suggesting that greater surveillance among exposed patients was unlikely to fully explain the observed associations. Subsequent low 25(OH)D levels during follow-up were substantially more common in the VDD group (HR, 3.62; *p* < 0.001), supporting the durability of the baseline exposure classification.

**TABLE 3:** Control and supportive outcome analyses after propensity score matching.

Outcome	VDD group (*n* = 135,329)	Control group (*n* = 135,329)	HR (95% CI)	*P*-value
	Events (%)	Events (%)		
Secondary hyperparathyroidism	483 (0.36)	314 (0.23)	1.50 (1.30–1.73)	<0.001
Acute appendicitis	378 (0.28)	336 (0.25)	1.08 (0.93–1.25)	0.298
Any follow-up healthcare encounter	123,887 (91.55)	126,370 (93.38)	0.92 (0.91–0.93)	<0.001
Subsequent VDD during follow-up	19,578 (14.47)	5,754 (4.25)	3.62 (3.51–3.73)	<0.001

Data are presented as number of events (%). Secondary hyperparathyroidism was used as a positive control outcome, whereas acute appendicitis was used as a negative control outcome. Follow-up healthcare encounters were assessed to examine potential surveillance bias, and subsequent VDD during follow-up was assessed to evaluate exposure durability. CI, confidence interval; HR, hazard ratio; VDD, vitamin D deficiency.

### 3.4 Sensitivity and subgroup analyses

The primary association remained stable across all four sensitivity models, with HRs ranging from 1.29 to 1.42 (all *p* < 0.001). The association with mortality also persisted in models in which mortality could be assessed, except for Model I, in which mortality was not applicable because the analysis was restricted to patients who survived throughout the follow-up. Intractable epilepsy consistently showed the largest relative estimates across the sensitivity models (HR range, 1.62–1.76) (Table 4).

**TABLE 4:** Sensitivity analyses for the association between vitamin D deficiency and risks of epilepsy-related outcomes.

Outcomes	Model I		Model II		Model III		Model IV	
	HR (95% CI)	*P-*value	HR (95% CI)	*P-*value	HR (95% CI)	*P-*value	HR (95% CI)	*P*-value
Epilepsy or seizure	1.29 (1.17–1.41)	<0.001	1.35 (1.25–1.46)	<0.001	1.32 (1.22–1.43)	<0.001	1.42 (1.28–1.58)	<0.001
Focal epilepsy	1.24 (1.02–1.50)	0.027	1.38 (1.17–1.62)	<0.001	1.38 (1.17–1.63)	<0.001	1.50 (1.21–1.86)	<0.001
Generalized epilepsy	1.40 (1.05–1.85)	0.020	1.40 (1.10–1.78)	0.006	1.30 (1.03–1.65)	0.025	1.57 (1.15–2.15)	0.004
Intractable epilepsy	1.68 (1.11–2.55)	0.013	1.75 (1.22–2.51)	0.002	1.62 (1.13–2.32)	0.008	1.76 (1.13–2.74)	0.011
Antiepileptics	1.24 (1.21–1.26)	<0.001	1.25 (1.22–1.27)	<0.001	1.23 (1.21–1.25)	<0.001	1.18 (1.15–1.21)	<0.001
Mortality	NA	NA	1.48 (1.44–1.52)	<0.001	1.48 (1.44–1.53)	<0.001	1.80 (1.74–1.87)	<0.001

Model I was restricted to patients who survived throughout follow-up. Model II was restricted to patients with at least one recorded healthcare encounter during follow-up. Model III excluded patients with a previous history of hypoglycemia. Model IV excluded patients whose vitamin D status crossed the prespecified exposure threshold during follow-up, defined as 25-hydroxyvitamin D ≥ 20 ng/mL in the VDD group or <30 ng/mL in the control group. CI, confidence interval; HR, hazard ratio; NA, not applicable; VDD, vitamin D deficiency.

In age-stratified analyses, the association with the primary outcome was stronger among patients older than 65 years (HR, 1.44, *p* < 0.001) than among those aged 18–65 years (HR, 1.15, *p* = 0.039), with a significant interaction (*p* for interaction = 0.006) (Table 5). Significant interactions were also observed for focal epilepsy and antiepileptic drug use (*p* for interaction = 0.026 and <0.001, respectively), with stronger estimates in the older stratum. In sex-stratified analyses (Table 6), the estimate for the primary outcome was numerically higher in women (HR, 1.49, *p* < 0.001) than in men (HR, 1.20, *p* = 0.004), although the interaction for the primary outcome did not reach statistical significance (*p* for interaction = 0.084). Significant interactions were observed for focal epilepsy and antiepileptic medication use (*p* for interaction = 0.006 for both), with stronger estimates in women.

**TABLE 5:** Age-stratified subgroup analyses for the association between vitamin D deficiency and risks of epilepsy-related outcomes.

Outcomes	18–65 years		>65 years		*p* for
	(*n* = 59,286 for each group)		(*n* = 75,041 for each group)		interaction
	HR (95% CI)	*P-*value	HR (95% CI)	*P-*value	
Epilepsy or seizure	1.15 (1.01–1.31)	0.039	1.44 (1.31–1.59)	<0.001	0.006
Focal epilepsy	1.05 (0.79–1.39)	0.760	1.54 (1.26–1.88)	<0.001	0.026
Generalized epilepsy	1.08 (0.72–1.63)	0.695	1.57 (1.17–2.10)	0.003	0.140
Intractable epilepsy	1.75 (1.02–3.02)	0.040	2.05 (1.26–3.32)	0.003	0.682
Antiepileptics	1.18 (1.15–1.22)	<0.001	1.28 (1.25–1.31)	<0.001	<0.001
Mortality	1.45 (1.34–1.57)	<0.001	1.46 (1.42–1.50)	<0.001	0.872

CI, confidence interval; HR, hazard ratio.

**TABLE 6:** Sex-stratified subgroup analyses for the association between vitamin D deficiency and risks of epilepsy-related outcomes.

Outcomes	Male		Female		*P* for interaction
	(*n* = 54,116 for each group)		(*n* = 80,855 for each group)		
	HR (95% CI)	*P-*value	HR (95% CI)	*P-*value	
Epilepsy or seizure	1.20 (1.06–1.37)	0.004	1.49 (1.34–1.64)	<0.001	0.084
Focal epilepsy	1.06 (0.83–1.37)	0.632	1.71 (1.37–2.12)	<0.001	0.006
Generalized epilepsy	1.25 (0.85–1.85)	0.257	1.44 (1.07–1.95)	0.017	0.576
Intractable epilepsy	1.49 (0.76–2.91)	0.242	1.99 (1.29–3.05)	0.001	0.481
Antiepileptics	1.20 (1.16–1.24)	<0.001	1.27 (1.24–1.30)	<0.001	0.006
Mortality	1.42 (1.37–1.48)	<0.001	1.48 (1.43–1.54)	<0.001	0.131

CI, confidence interval; HR, hazard ratio.

### 3.5 Multivariable cox regression

Within the entire unmatched cohort (Table 7), the link between VDD and incident epilepsy or seizure persisted even after adjusting for demographic and clinical covariates concurrently (HR, 1.41, *p* < 0.001). The strongest covariate associations were cerebrovascular disease (2.61, *p* < 0.001) and alcohol-related disorders (HR, 2.26, *p* < 0.001), followed by a recorded glucose level <70 mg/dL (HR, 1.93, *p* < 0.001), chronic kidney disease (HR, 1.37, *p* < 0.001), nicotine dependence (HR, 1.31, *p* < 0.001), hemoglobin A1c ≥ 9% (HR, 1.16, *p* = 0.003), and ischemic heart disease (HR, 1.14, *p* = 0.008). In contrast, disorders of lipoprotein metabolism (HR, 0.76, *p* < 0.001) and overweight or obesity (HR, 0.88, *p* = 0.002) were inversely associated with the outcome.

**TABLE 7:** Multivariable Cox regression model for incident epilepsy or seizure in the unmatched cohort.

Variable	HR (95% CI)	*P-*value
VDD vs. control group	1.41 (1.31–1.51)	<0.001
Male	0.95 (0.88–1.02)	0.145
Age at index	1.01 (1.01–1.01)	<0.001
White	1.02 (0.95–1.10)	0.594
Essential hypertension	0.99 (0.91–1.08)	0.788
Disorders of lipoprotein metabolism and other lipidemias	0.76 (0.70–0.82)	<0.001
Overweight and obesity	0.88 (0.81–0.95)	0.002
Chronic kidney disease	1.37 (1.24–1.51)	<0.001
Ischemic heart diseases	1.14 (1.04–1.25)	0.008
Nicotine dependence	1.31 (1.18–1.47)	<0.001
Cerebrovascular diseases	2.61 (2.36–2.88)	<0.001
Alcohol-related disorders	2.26 (1.91–2.67)	<0.001
Type 2 diabetes mellitus with hypoglycemia	1.16 (0.90–1.48)	0.258
Glucose < 70 mg/dL	1.93 (1.73–2.14)	<0.001
Hemoglobin A1c ≥ 9%	1.16 (1.05–1.28)	0.003
Diseases of liver	1.07 (0.95–1.22)	0.275

CI, confidence interval; HR, hazard ratio; VDD, vitamin D deficiency.

### 3.6 Exposure-gradient analysis

Across vitamin D categories, a graded association was observed for the primary outcome of epilepsy or seizure, with progressively higher estimates from vitamin D insufficiency to VDD and severe VDD, with HRs of 1.23, 1.37, and 1.44, respectively (all *p* < 0.001) (Table 8). Similar graded patterns were observed for focal epilepsy (HRs 1.13, 1.40, and 1.63), intractable epilepsy (HRs 1.85, 1.92, and 2.38), antiepileptic medication use (HRs 1.18, 1.25, and 1.34), and mortality (HRs 1.19, 1.47, and 1.67). In contrast, the gradient was less consistent for generalized epilepsy, for which the estimates of vitamin D insufficiency and VDD were similar. Overall, these exploratory findings suggest a graded association with most epilepsy-related outcomes, except generalized epilepsy.

**TABLE 8:** Exposure-gradient analysis comparing severe vitamin D deficiency, vitamin D deficiency, and vitamin D insufficiency with normal vitamin D status.

Outcomes	Severe VDD (*n* = 41,597 for each group)		VDD (primary analysis) (*n* = 135,329 for each group)		VDI (*n* = 185,674 for each group)	
	HR (95% CI)	*P-*value	HR (95% CI)	*P-*value	HR (95% CI)	*P-*value
Epilepsy or seizure	1.44 (1.27–1.64)	<0.001	1.37 (1.26–1.48)	<0.001	1.23 (1.15–1.32)	<0.001
Focal epilepsy	1.63 (1.25–2.13)	<0.001	1.40 (1.19–1.65)	<0.001	1.13 (0.98–1.31)	0.087
Generalized epilepsy	1.63 (1.14–2.33)	0.006	1.42 (1.13–1.80)	0.003	1.42 (1.15–1.76)	0.001
Intractable epilepsy	2.38 (1.36–4.18)	0.002	1.92 (1.33–2.76)	<0.001	1.85 (1.33–2.58)	<0.001
Antiepileptics	1.34 (1.30–1.39)	<0.001	1.25 (1.23–1.27)	<0.001	1.18 (1.16–1.20)	<0.001
Mortality	1.67 (1.59–1.75)	<0.001	1.47 (1.43–1.51)	<0.001	1.19 (1.16–1.22)	<0.001

Severe VDD was defined as 25-hydroxyvitamin D < 10 ng/mL, VDD as <20 ng/mL, vitamin D insufficiency as 20–29.9 ng/mL, and normal vitamin D status as ≥30 ng/mL. These analyses were exploratory, and no formal trend test was performed. CI, confidence interval; HR, hazard ratio; VDD, vitamin D deficiency; VDI, vitamin D insufficiency.

## 4 Discussion

In this large multinational propensity score–matched cohort of adults with T2DM, VDD was associated with a higher long-term risk of incident epilepsy or seizure. The association was observed across epilepsy subtypes and was most pronounced for intractable epilepsy, a clinically severe phenotype. The primary association remained robust across sensitivity analyses addressing differential mortality, follow-up ascertainment, prior hypoglycemia, and time-varying vitamin D status. Most epilepsy-related outcomes also showed progressively higher risk estimates across lower vitamin D categories. The prespecified positive and negative control outcomes behaved as expected. In addition, the primary association remained consistent in the sensitivity analysis restricted to patients with at least one recorded follow-up encounter. However, despite the statistically significant relative association, the absolute incidence difference was small (1.10% vs. 0.78%; absolute difference, 0.32 percentage points). Accordingly, the clinical relevance of this finding should be interpreted cautiously, and VDD should be regarded primarily as a potential risk marker rather than an indicator of a large absolute increase in seizure risk.

These findings extend the prior literature, which has primarily focused on the reverse direction of association between epilepsy and vitamin D status. Previous studies have frequently reported a high prevalence of VDD among patients with established epilepsy, often attributed to long-term antiseizure medication use, reduced mobility, and limited sunlight exposure (18–20). However, these cross-sectional or treatment-focused studies cannot establish whether low vitamin D status temporally precedes the development of incident epilepsy or seizure. By excluding patients with pre-existing epilepsy or antiseizure medication use and applying a 1-year landmark design, the present study specifically evaluated the temporal relationship between antecedent VDD and subsequent incident epilepsy or seizure in adults with T2DM, a population in which both VDD and seizure-related risk factors are common.

The multivariable Cox analysis supported the internal coherence of the findings by reproducing associations with established seizure-related risk factors. Consistent with the established epidemiology of seizure-related outcomes in diabetes and older adults, cerebrovascular disease, recorded glucose <70 mg/dL, and older age were independently associated with incident epilepsy or seizure (5, 12, 28). These findings are clinically plausible because cerebrovascular injury and hypoglycemia are well-recognized seizure precipitants, particularly in patients with metabolic vulnerability. Interestingly, disorders of lipoprotein metabolism and being overweight or obese were inversely associated with incident epilepsy or seizure in our unmatched multivariable model. A similar pattern has been reported previously; in a study of older veterans, Pugh et al. (28) similarly reported that hypercholesterolemia and obesity were associated with a lower likelihood of new-onset epilepsy, whereas cerebrovascular disease was strongly associated with an increased risk. Therefore, the inverse associations observed in our study should be interpreted cautiously rather than as evidence of a protective biological effect. Potential explanations include residual confounding, differences in treatment patterns such as statin use, healthcare behavior, diagnostic coding, survival or competing risk mechanisms, and collider bias. Overall, the multivariable findings support the coherence of the VDD–epilepsy association with recognized seizure-related risk factors while also highlighting the complexity of interpreting metabolic covariates in observational EHR-based research.

Several hypothesized mechanisms may provide biological plausibility for the observed association, as vitamin D signaling has been implicated in neuronal calcium regulation, neurotrophin expression, oxidative stress, and inflammatory pathways that may be relevant to neuronal excitability (29–31). In patients with T2DM, these mechanisms could interact with pre-existing metabolic, vascular, and inflammatory vulnerability; however, these pathways were not directly evaluated in the present study and remain speculative. The exposure-gradient findings provided additional supportive evidence but should be interpreted as exploratory because no formal trend test was performed. Most epilepsy-related outcomes, including the primary outcome, focal epilepsy, intractable epilepsy, and antiepileptic medication use, showed progressively higher estimates from vitamin D insufficiency to VDD and severe VDD. However, this pattern was not fully consistent for generalized epilepsy, and the analysis should be interpreted with caution. Because the TriNetX platform does not support patient-level continuous exposure modeling or formal trend testing, we could not perform a continuous dose-response analysis or formally test linearity or nonlinearity. Therefore, the observed pattern should be regarded strictly as hypothesis-generating and should not be interpreted as evidence of a statistically confirmed dose-response relationship.

The apparent discrepancy between our findings and those of Mendelian randomization studies warrants careful interpretation. Genetic studies have generally reported null or inconsistent associations between genetically predicted 25(OH)D levels and epilepsy risk (21–23), suggesting that the direct lifelong causal effect of vitamin D on epilepsy may be limited or difficult to detect. However, Mendelian randomization and real-world cohort studies address different research questions. Genetically predicted 25(OH)D in Mendelian randomization studies represents lifelong genetic liability to circulating 25(OH)D levels, whereas clinically measured VDD may reflect an acquired and dynamic risk state influenced by sunlight exposure, nutrition, adiposity, inflammation, renal dysfunction, metabolic control, and comorbidity burden. Therefore, null or inconsistent genetic evidence does not necessarily exclude a clinically relevant association between VDD and subsequent epilepsy or seizure in patients with T2DM. The contribution of the present study is to evaluate this clinically relevant temporal relationship in a large T2DM population, rather than to establish causality. This study provides real-world evidence that VDD may serve as a risk marker for subsequent epilepsy or seizure in patients with T2DM. These findings should be interpreted as hypothesis-generating and complementary to genetic evidence, not as proof that vitamin D supplementation prevents epilepsy.

From a clinical perspective, serum 25(OH)D testing is widely available, and vitamin D status is routinely measurable and potentially modifiable in patients with T2DM. Although the economic burden of testing and treatment varies across countries and healthcare systems, serum 25(OH)D measurement is generally minimally invasive and clinically accessible. However, the present findings should not be interpreted as evidence that vitamin D supplementation prevents epilepsy or seizure. Rather, clinically measured VDD may help identify patients with a higher burden of metabolic, vascular, inflammatory, or nutritional vulnerability who may warrant closer clinical attention to potential neurological complications. Clinical trials in patients with established epilepsy have begun to examine whether correction of VDD can improve seizure-related outcomes, but the findings remain preliminary and have not established a preventive effect in individuals without epilepsy (32, 33). Future prospective studies should determine whether targeted or periodic vitamin D screening improves risk identification, whether correction of VDD modifies seizure-related outcomes, and whether such strategies are clinically effective and cost-effective. These studies should also establish the appropriate screening interval and identify subgroups, such as older adults or patients with severe or persistent VDD, who may derive the greatest benefit. Until such evidence is available, universal annual vitamin D screening solely for epilepsy prevention cannot be recommended.

Several limitations should be considered. First, residual confounding is unavoidable in observational EHR studies because several potentially relevant factors were unavailable, including sunlight exposure, seasonality, dietary habits, physical activity, socioeconomic status, vitamin D supplementation and adherence to supplementation, diabetes duration and severity, and adherence to other medications. Poorer lifestyle, socioeconomic, or diabetes-related profiles in the VDD cohort could have exaggerated the observed association, whereas supplementation or favorable behavioral changes after baseline could have attenuated it. Therefore, the direction and magnitude of the remaining residual confounding are uncertain. Second, exposure misclassification may have occurred because vitamin D status was based on the first qualifying 25(OH)D measurement and could subsequently have changed owing to supplementation, seasonal variation, or lifestyle changes. The 5-year look-back classification, assessment of exposure durability during follow-up, and sensitivity analysis excluding patients whose vitamin D status crossed the predefined threshold partially mitigated this concern but could not replace a formal repeated-measure analysis. Information on vitamin D replacement therapy, including dose, duration, adherence, and over-the-counter supplementation, was also unavailable; treatment during follow-up may therefore have attenuated or otherwise modified the observed associations. Third, outcome misclassification is possible because epilepsy, seizure, and epilepsy subtypes were identified using ICD-10-CM codes without confirmation by EEG findings, neurologist assessment, neuroimaging, or chart review. Although previous validation studies (34, 35) have shown generally acceptable accuracy for identifying epilepsy from administrative data, diagnostic performance varies across healthcare settings and may be less consistent for specific epilepsy subtypes. Moreover, we could not perform validation specifically within TriNetX. Therefore, some outcome misclassification remains possible, and its direction and magnitude cannot be determined. Fourth, selection bias may arise because eligibility required a documented 25(OH)D measurement. Tested patients may have greater comorbidity burden or healthcare utilization than the broader T2DM population, and propensity score matching could not account for unmeasured determinants of testing. Therefore, the findings are most applicable to patients with T2DM who undergo vitamin D testing. Fifth, unexpected inverse associations between dyslipidemia and obesity suggest possible residual confounding, collider bias, or competing risk mechanisms. Sixth, mortality was higher in the VDD cohort and may have competed with the occurrence or ascertainment of epilepsy or seizure. Because the cause-specific Cox model does not directly estimate cumulative incidence in the presence of competing mortality, the reported HRs should be interpreted accordingly. Although the survivor-restricted sensitivity analysis yielded consistent findings, it could not fully substitute for a formal competing-risk analysis. Finally, subgroup, sensitivity, and exposure-gradient analyses were exploratory and uncorrected for multiplicity, increasing the possibility of chance findings. Moreover, the absence of a formal trend test limits interpretation of the exposure-gradient results as evidence of a dose-response relationship. The generalizability of these findings is limited because the study included only adults with T2DM who had documented 25(OH)D measurements. The results may therefore not apply to individuals without diabetes, pediatric populations, or patients with T2DM who do not undergo vitamin D testing.

## 5 Conclusion

Among adults with T2DM, clinically measured VDD was associated with a higher long-term risk of incident epilepsy or seizure. The strongest relative estimate was observed for intractable epilepsy, and risk estimates generally increased across progressively lower vitamin D categories. These findings suggest that VDD may serve as a clinically relevant risk marker in this population, but they do not establish a causal relationship or demonstrate that vitamin D supplementation can prevent epilepsy or seizure.

## Data Availability

The datasets presented in this article are not readily available because the datasets used in this study were obtained from the TriNetX Global Collaborative Network. Due to data use agreements, privacy protections, and licensing restrictions, the underlying patient-level data cannot be shared by the corresponding author. Access to the data is restricted to authorized users through the TriNetX platform. The study used de-identified electronic health record data, and only aggregated results generated from the platform are reported in the manuscript. Requests to access the datasets should be directed to https://live.trinetx.com.
