# Supplementary material for: Vitamin D deficiency and long-term risk of epilepsy or seizure in type 2 diabetes: a multicenter real-world cohort study
Source: Front Nutr. 2026 Aug 7;13:1910295. doi: 10.3389/fnut.2026.1910295 (PMC13495779; doi:10.3389/fnut.2026.1910295)
Supplement: Supplementary file 1 [file Table_1.docx]

**Supplemental Table 1. Codes used to define exclusion criteria**

| Exclusion criterion | Code / concept |
| --- | --- |
| Pre-existing epilepsy or recurrent seizures | ICD-10-CM: G40 |
| Prior antiepileptic medication use | ATC: N03A |
| Death during landmark period | Deceased; ICD-10-CM: R99 |
| End-stage renal disease | ICD-10-CM: N18.6 |
| Chronic kidney disease, stage 4 | ICD-10-CM: N18.4 |
| Chronic kidney disease, stage 5 | ICD-10-CM: N18.5 |
| Dependence on renal dialysis | ICD-10-CM: Z99.2 |
| HIV disease | ICD-10-CM: B20 |
| Asymptomatic HIV infection status | ICD-10-CM: Z21 |
| Liver fibrosis or cirrhosis | ICD-10-CM: K74 |
| Celiac disease | ICD-10-CM: K90.0 |
| Crohn’s disease | ICD-10-CM: K50 |
| Short bowel syndrome | ICD-10-CM: K90.82 |
| Other pancreatic diseases | ICD-10-CM: K86 |
| Pregnancy | ICD-10-PCS: 10; ICD-10-CM: Z33.1; LOINC: 82810-3 |
| Critical care service use | CPT: 1013729 |
| Acute kidney failure | ICD-10-CM: N17 |
| Severe sepsis | ICD-10-CM: R65.2 |
| Other sepsis | ICD-10-CM: A41 |
| Intracranial injury | ICD-10-CM: S06 |
| Malignant brain neoplasm | ICD-10-CM: C71 |
| Benign neoplasm of brain or CNS | ICD-10-CM: D33 |
| Encephalitis, myelitis, and encephalomyelitis | ICD-10-CM: G04 |
| Anoxic brain damage | ICD-10-CM: G93.1 |
| Cerebral palsy | ICD-10-CM: G80 |
| Vascular dementia | ICD-10-CM: F01 |
| Alzheimer’s disease | ICD-10-CM: G30 |
| Unspecified dementia | ICD-10-CM: F03 |
| Dementia in other diseases classified elsewhere | ICD-10-CM: F02 |
| Prior non-deficient vitamin D level in VDD cohort | 25-hydroxyvitamin D ≥20 ng/mL |
| Prior low/insufficient vitamin D level in control cohort | 25-hydroxyvitamin D <30 ng/mL |

**Supplemental Table 2. Codes for variables used in propensity score matching**

| Variable | Code / term used in TriNetX |
| --- | --- |
| Factors influencing health status and contact with health services | Z00–Z99 |
| Essential hypertension | I10 |
| Disorders of lipoprotein metabolism and other lipidemias | E78 |
| Overweight and obesity | E66 |
| Neoplasms | C00–D49 |
| Ischemic heart diseases | I20–I25 |
| Obstructive sleep apnea | G47.33 |
| Anemias | D64 |
| Chronic kidney disease | N18 |
| Nicotine dependence | F17 |
| Diseases of liver | K70–K77 |
| Chronic obstructive pulmonary disease | J44 |
| Cerebrovascular diseases | I60–I69 |
| Type 2 diabetes mellitus with kidney complications | E11.2 |
| Type 2 diabetes mellitus with neurological complications | E11.4 |
| COVID-19 | U07.1 |
| Type 2 diabetes mellitus with ophthalmic complications | E11.3 |
| Alcohol-related disorders | F10 |
| Type 2 diabetes mellitus with hypoglycemia | E11.64 |
| Malnutrition | E40–E46 |
| Metformin | 6809 |
| Insulins and analogues | A10A |
| Vitamin D supplementation | VT500 |
| Glucagon-like peptide-1 receptor agonists | A10BJ |
| Sodium-glucose cotransporter 2 inhibitors | A10BK |
| Glucose <70 mg/dL | 9025, Glucose [Mass/volume] in Serum, Plasma or Blood |
| Hemoglobin >12 g/dL | 9014, Hemoglobin [Mass/volume] in Blood |
| Albumin >3.5 g/dL | 9045, Albumin [Mass/volume] in Serum, Plasma or Blood |

**Supplemental Table 3. Definitions of primary, secondary, control, and supportive outcomes used in the TriNetX analysis.**

| Outcome category | Outcome | Definition / code used in TriNetX |
| --- | --- | --- |
| Primary outcome | Epilepsy or seizure | Epilepsy and recurrent seizures; ICD-10-CM G40 |
| Secondary outcome | Focal epilepsy | Localization-related focal epilepsy; ICD-10-CM G40.0–G40.2 |
| Secondary outcome | Generalized epilepsy | Generalized epilepsy and epileptic syndromes; ICD-10-CM G40.3–G40.4 |
| Secondary outcome | Intractable epilepsy | Epilepsy, unspecified, intractable; ICD-10-CM G40.91 |
| Secondary outcome | Antiepileptic medication use | Antiepileptics; ATC N03A |
| Secondary outcome | All-cause mortality | Deceased status or ill-defined/unknown cause of mortality; Deceased; ICD-10-CM R99 |
| Positive control outcome | Secondary hyperparathyroidism | Secondary hyperparathyroidism, not elsewhere classified; ICD-10-CM E21.1 |
| Negative control outcome | Acute appendicitis | Acute appendicitis; ICD-10-CM K35 |
| Supportive outcome | Any follow-up healthcare encounter | Any visit during follow-up; TriNetX visit record |
| Supportive outcome | Subsequent low 25-hydroxyvitamin D during follow-up | Follow-up 25(OH)D below the predefined threshold; TriNetX laboratory record / TNX:9034 |
